# Chemical, antimicrobial, and molecular characterization of mortiño (*Vaccinium floribundum* Kunth) fruits and leaves

**DOI:** 10.1002/fsn3.638

**Published:** 2018-03-26

**Authors:** Susana Llivisaca, Patricia Manzano, Jenny Ruales, José Flores, Joffre Mendoza, Esther Peralta, Juan M. Cevallos‐Cevallos

**Affiliations:** ^1^ Centro de Investigaciones Biotecnológicas del Ecuador (CIBE) Escuela Superior Politécnica del Litoral (ESPOL) Guayaquil Ecuador; ^2^ Facultad Ciencias de la Vida (FCV) Escuela Superior Politécnica del Litoral (ESPOL) Guayaquil Ecuador; ^3^ Departamento de Ciencia de Alimentos y Biotecnología (DECAB) Escuela Politécnica Nacional (EPN) Quito Ecuador

**Keywords:** anthocyanins, antioxidants, matK, polyphenols, rbcL

## Abstract

Fruits and leaves of *Vaccinium* spp. are known for their high content of bioactive compounds, but the chemical and biological characteristics of mortiño (*Vaccinium floribundum* Kunth) have not been fully described. In this study, the levels of polyphenols, antioxidant capacity, anthocyanins, antimicrobial activity, and genetic variability were determined in mortiño plants. The Folin–Ciocalteu's, ABTS scavenging, pH differential, and well diffusion methods were used to evaluate the levels of polyphenols, antioxidant capacity, anthocyanins, and antimicrobial activity, respectively. The genetic variability was evaluated by sequencing of the matK and rbcl DNA regions. Polyphenol content was up to 229.81 mg gallic acid equivalents/100 g, the average antioxidant capacity was 11.01 mmol Trolox equivalents/100 g, and anthocyanin content was up to 1,095.39 mg/100 g. Mortiño extracts significantly inhibited the growth of Gram‐negative bacteria including *Burkholderia gladioli*,* Burkholderia cepacia*,* Salmonella* Typhimurium, *Vibrio parahaemolyticus*,* Vibrio harveyi*,* Vibrio vulnificus*,* Escherichia coli*, and *Pseudomona aeruginosa*, as well as Gram‐positive bacteria such as *Probionibacterium propionicum*,* Staphylococcus aureus*, and *Enterococcus faecalis* showing greater inhibition halos than those produced by the antibiotic ampicillin. A polymorphic nucleotide was found in position 739 of the matK region. This study shows the potential of mortiño for the food and pharmaceutical industries.

## 1 INTRODUCTION

The genus *Vaccinium* comprises several important crops of the temperate zone, including *V. corymbosum*,* V. ashei*, and *V. angustifolium* (Medina Cano, Lobo Arias, Castaño Colorado, & Cardona, 2015). Plants of *Vaccinium floribundum* Kunth—aka mortiño—grow spontaneously in Ecuador's Andean highlands (Prencipe et al., 2014) producing a sweet fruit with pleasant flavor that is mostly used in jams, wine, and boiled drinks (Ortiz, Marín‐Arroyo, Noriega‐Domínguez, Navarro, & Arozarena, 2013). The fruits of *Vaccinium* spp. such as blueberries and cranberries are generally known as “superfruits” because of their high concentration of bioactive compounds including polyphenols, anthocyanins, and antioxidants (Howell, 2009), but the levels of bioactive compounds in mortiño plants have not been fully characterized.

Molecular characterization of plant species is crucial to avoid identification errors and infer relationships between organisms (Borsch & Quandt, 2010). Molecular characterization of plants has frequently been carried out by sequencing of the matK and rbcL DNA regions (Hilu, 1997). However, identification of mortiño plants has mostly relied on the observation of taxonomic traits without any molecular confirmation (Pérez Flores & Valdivieso Noguera, 2007). Moreover, no rcbL DNA sequences of this plant species could be found in databases prior to this study.

Recent reports showed that the fruits of mortiño may contain substantial amounts of sugars, antioxidants, vitamins B and C, minerals, and anthocyanins (Lee, 2016). However, studies on polyphenolic compounds, anthocyanins, antioxidant capacity, and antimicrobial activity on leaves of *V. floribundum* cannot be found in the literature. The characterization of mortiño leaves has the potential to introduce a novel viable source of pharmaceutical products (Ortiz et al., 2013).

Extracts of *Vaccinium* spp. have shown significant antimicrobial properties. Extracts of *V. corymbosum* were able to inhibit the growth of several pathogens including *Salmonella* spp. (Pervin, Hasnat, & Lim, 2013) and *Listeria monocytogenes* (Shen et al., 2014). Similarly, *Vaccinium macrocarpon* extracts caused the inhibition of *Bacillus cereus* and *Micrococcus luteus* (Viskelis et al., 2009). However, the antimicrobial properties of mortiño fruits and leaves have not been described.

The objective of this study was to determine the contents of polyphenols, antioxidants, and anthocyanins and assess the antimicrobial potential of mortiño leaves and fruits. Characterization of matK and rbcL DNA regions was also performed.

## 2 MATERIALS AND METHODS

### 2.1 Collection and preparation of samples

Mortiño plants of approximately 1.5 m of height were collected from the tundra of the Ecuadorian provinces of Pichincha, Cotopaxi, and Chimborazo. At least 10 plants were collected from each sampled province. Due to difficulties in geographical accessibility, samples from Cotopaxi were only used for molecular analysis. All plants were initially identified by a local guide, and the ID was confirmed by molecular methods as described in Section 2.2. About 50 g of fruit and leaf samples was taken from each plant, but deteriorated fruit samples were discarded. All samples were rinsed with approximately 500 ml of distilled water and lyophilized on a LYOVAC GT2 freeze‐dryer (GEA Group, Düsseldorf, Germany) prior to analysis.

### 2.2 Genetic characterization

The DNA was extracted from leaf samples exactly as described elsewhere (Azmat et al., 2012), and PCRs were carried out using the primers matK_3f_KIM_f CGTACAGTACTTTTGTGTTTACGAG and matK_1r_KIM_r ACCCAGTCCATCTGGAAATCTTGGTTC for the matK DNA region, as well as rbcL_F ATGTCACCACAAACAGAGACTAAAGC and rbcL_R GTAAAATCAAGTCCACCRCG for the rbcL DNA region. All PCRs were run using 1 μl of Go‐Taq Green Master Mix (Promega, USA) at 2×, 2 μl of DNA, and 0.5 μl of each forward and reverse primer at 20 ng/μl. Distilled water was added to a final reaction volume of 10 μl. The thermocycler (Eppendorf, nexus GSX1, Spain) program consisted of an initial denaturation at 95°C for 1 min followed by 35 cycles of denaturing at 95°C, annealing at 50°C, and extension at 72°C, followed by a final extension step at 72°C for 1 min (Cobo, Gutiérrez, Torres, & Torres, 2016). The PCR products were then sent for sequencing to a service laboratory, and the resulting sequences were compared with those in the NCBI database using BLAST. A neighbor‐joining phylogenetic tree was constructed with 1,000 bootstrap replicates using GENEIOUS (García, Ligarreto, and Adolfo, 2014), and polymorphic nucleotides were estimated by aligning all matK and rbcL DNA consensus sequences using the MUSCLE algorithm in MEGA 7.0 (Tamura, Stecher, Peterson, Filipski, & Kumar, 2013).

### 2.3 Total polyphenol content

Total polyphenol content was estimated according to the Folin–Ciocalteu's method (Ortiz et al., 2013). Briefly, 10 ml of 70% acetone was added to 0.2 g of lyophilized sample and liquefied in a blender (Oster Super Deluxe, USA) for 10 min followed by incubation at 24°C for 10 min. The extracts were then filtered in Whatman number 41 papers and stored in amber flasks at 4°C. About 0.5 ml of each extract was diluted with 3.5 ml of distilled water and filtered through an OASIS cartridge (Oasis HLB 6 cc Vac Cartridge; Cod. WAT106202; USA) to remove interfering compounds. About 500 μl of the filtrate was then mixed with 2.5 ml of 2 N Folin–Ciocalteu reagent (Sigma‐Aldrich, Germany), homogenized in a vortex apparatus (Boeco brand, model V1 PLUS), and allowed to stand for 2 min at room temperature. The solution was then combined with 2 ml of a 7.5% Na_2_CO_3_ solution and incubated in a water bath at 50°C for 15 min. The solution was rapidly cooled in a 4°C ice bath to stop the reaction, and the absorbance was measured at 760 nm in a spectrophotometer (Shimadzu Uv‐160 Serial No: 29 D0670; Japan). All samples were prepared and analyzed in triplicated. The results were expressed as gallic acid equivalents (GAEs) using a calibration curve built with the pure standard over the range of 10–100 ppm.

### 2.4 In vitro antioxidant capacity

The 2,2′‐azino‐bis‐3‐ethylbenzthiazoline‐6‐sulfonic acid^+^ (ABTS^+^) scavenging ability was determined using the method described in a previous report (Thaipong, Boonprakob, Crosby, Cisneros‐Zevallos, & Hawkins Byrne, 2006). Briefly, 10 ml of 70% acetone was added to 0.2 g of lyophilized sample and liquefied in a blender (Oster Super Deluxe, USA) for 10 min followed by incubation at 24°C for 10 min. The extracts were then filtered in Whatman number 41 papers and stored in amber flasks at 4°C. Meanwhile, about 96 mg of ABTS (Sigma‐Aldrich, USA) was mixed with 16 mg of potassium peroxide disulfate (Sigma‐Aldrich, Germany) plus 25 ml of distilled water and allowed to react in darkness at room temperature for 24 hr to form ABTS^+^. The ABTS^+^ solution was diluted to an absorbance of 0.7 ± 0.02 at 734 nm using ethanol. Extracts (10 μl) were added to 1 ml of ABTS^+^, and absorbance was measured at 734 nm after 1 and 6 min, respectively. The ABTS reduction was calculated using the equation below, and the results were expressed as mmol Trolox equivalent (TE) per 100 g of sample using a Trolox standard curve built in the range of 12.5%–100%.

$$\text{Cn}=\frac{\frac{\%I-b}{m}*V*\text{df}}{W}.$$

where, Cn is the concentration of TEs, *I* is the percentage of inhibition, df is the dilution factor, *V* is the volume of the extract, *W* is the weight of the sample, *b* is the intercept of the *Y* axis, and *m* is the slope.

### 2.5 Determination of total anthocyanins

The content of anthocyanins was determined by spectrophotometry using the pH differential method exactly as described elsewhere (Sarkis, Tessaro, & Marczak, 2013). For this, two sample suspensions were prepared by combining 1.5 g of lyophilized sample with 500 ml of either 0.2 mol/L hydrochloric acid‐potassium chloride buffer (pH 1.0) or 1 mol/L sodium acetate buffer (pH 4.5), respectively. Suspensions were then homogenized and passed through filter paper until a clear solution was obtained. Absorbance was then measured at 520 and 700 nm (Shimadzu UV‐160A, Kyoto, Japan) in each solution, and the anthocyanin content was estimated using the following equation (Jiang, Yang, & Shi, 2017):

$$\text{Anthocyanins content (mg/g)}=\frac{A\;\times \;\text{MW}\;\times \;\text{DF}\;\times \;V\;\times \;10^{3}}{\varepsilon \;\times \;l\times \;M},$$

where *A* = ((Absorbance_520 _− Absorbance_700_)_pH 1.0 _− (Absorbance_520 _− Absorbance_700_)_pH 4.5_), MW = molecular weight of cyanidin‐3‐glucoside (449.2 g/mol), DF = dilution factor, *V* = volume of the aliquot, ɛ = molar extinction coefficient of cyanidin‐3‐glucoside (29,600), *l* = path length, and *M* = weight of sample (g). Results were expressed as cyanidin‐3‐glucoside equivalents. All samples were analyzed in triplicate.

### 2.6 Antibacterial properties

Bacterial pathogens including *Burkholderia gladioli*;* Burkholderia cepacia*,* Enterococcus faecalis*;* Salmonella* Typhimurium; *Vibrio parahemolíticus*,* Probionibacterium propionicum*,* Staphylococcus aureus*, and *Pseudomonas aeruginosa* were grown in Luria–Bertani (LB) and Tryptic Soy Agars (TSAs) and incubated at 37°C. All pathogens were previously identified by sequencing of the 16s rDNA region and by biochemical tests (API 20 NE) and are part of our culture collection of microorganisms. After 24‐hr incubation, while in the exponential phase of growth to avoid the selection of atypical variants (Balouiri, Sadiki, & Ibnsouda, 2016), isolates were transferred to a 0.8% saline solution to a final bacterial concentration of 10^5 ^CFU/ml or 0.5 turbidity on the McFarland scale measured in a turbidimeter (Biomerieux, Densimat, France) as suggested in previous reports (Shen et al., 2014). Meanwhile, about 500 mg of each lyophilized sample from mortiño fruits or leaves was suspended in 100 ml of 70% ethanol, allowed to soak for 1 hr, and filtered through Whatman 41 paper (Balouiri et al., 2016). Each pathogen suspension was spread‐inoculated over the surface of TSA as well as LB and let air dry. Four wells of three millimeter diameter were made in the inoculated media using a sterile puncher, and 50 μl of each sample extract was added into each well. Ampicillin (50 μg/ml) was used as positive control, and 70% ethanol was used as a blank sample (Figure 5a,b). Plates were then incubated for 24 hr at 37°C, and the antibacterial activity was expressed as the zone of inhibition diameter (ZOI) produced by the extracts against the test bacteria. (Pervin et al., 2013) Five replicates were run for each pathogen.

### 2.7 Statistical analysis

Data of polyphenolic compounds, antioxidant capacity, anthocyanins, and antimicrobial activity were analyzed using Tukey's tests in Minitab 16 (Zhang, Yue, Jiang, Fan, & Gao, 2016), and significance was reported at *p* < .05. Correspondence analysis was carried out using InfoStat version 20161 to determine potential correlations between the bacterial Gram staining, potential degree of antibiotic resistance, and inhibition halo. For this, pathogens were first classified according to the antibiotic resistance potential into low, medium, high, or critically resistant to antibiotics following the WHO classification (WHO, 2014). Additionally, bacterial inhibition halos were divided into quartiles to obtain the low, medium–low, medium–high, and high halo size ranges.

## 3 RESULTS

### 3.1 Genetic characterization

**Figure 1:**
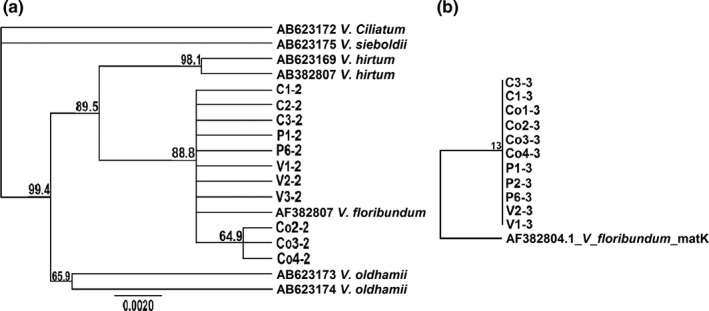
Neighbor‐joining tree of the matK (a) and rbcl (b) DNA regions. Samples were from Pichincha (P and V), Chimborazo (C), and Cotopaxi (Co) provinces. Bootstrap values were estimated from 1,000 replicates. Sequences of other Vaccinium spp. (a) and the matK region of V. floribundum (b) were used as outgroups

A total of 20 representative mortiño samples were sequenced, and four characteristic sequences of matK and one of the rbcL DNA regions were uploaded to the NCBI database (Table 1). Prior to this study, no rbcL and only one matK sequence from *Vaccinium floribundum* Kunth could be found in available databases. Sequences of the matK region shared a 100% similarity with the only mortiño accession in the database (accession AF382804), and the phylogenetic tree showed a subgroup formed by samples from the Cotopaxi province exclusively (Figure 1a). One polymorphic nucleotide in the matK region was found at position 739 as Cotopaxi sequences contained a G (guanine) instead of the C (cytosine) observed in the rest of the sequences at this position. No polymorphism was found in rbcL sequences, and all samples grouped in the same clade (Figure 1b).

**Table 1:** Accession numbers of the matK and rbcL sequences from mortiño

Accession numbers	Descriptions
KP973414	*Vaccinium floribundum* isolate C1‐2_A04 maturase K (matK) gene, partial cds; chloroplast
KP973415	*V. floribundum* isolate Co2‐2_A08 maturase K (matK) gene, partial cds; chloroplast
KP973416	*V. floribundum* isolate P1‐2_A01 maturase K (matK) gene, partial cds; chloroplast
KP973417	*V. floribundum* isolate V1‐2_A11 maturase K (matK) gene, partial cds; chloroplast
KP973418	*V. floribundum* isolate C1‐3_B04 ribulose‐1,5‐bisphosphate carboxylase/oxygenase large‐subunit (rbcL) gene, partial cds; chloroplast

### 3.2 Chemical characterization

**Figure 2:**
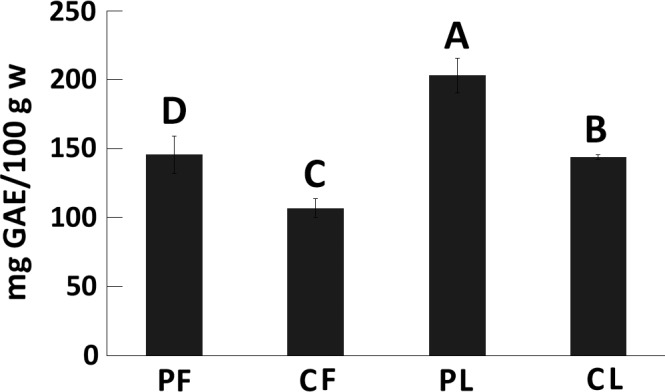
Polyphenol content of mortiño leaves and fruits from Pichincha (PF and PL) and Chimborazo (CF, and CL). Different letters (A, B, C, and D) above each column represent significant differences (*p* < .05) in the average polyphenol content of each sample group

**Figure 3:**
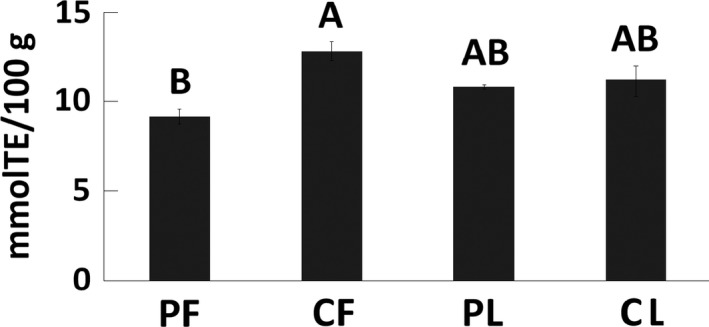
Antioxidant capacity of mortiño leaves and fruits from Pichincha (PF and PL) and Chimborazo (CF, and CL). Different letters (A and B) above each column represent significant differences (*p* < .05) in the average antioxidant capacity of each sample group

Mortiño samples from Pichincha yielded significantly higher concentrations of polyphenols (146.10 ± 13.44 and 204.01 ± 12.50 mg GAEs/100 g for fruits and leaves, respectively) than the samples from Chimborazo (107.37 ± 6.71 and 144.33 ± 1.85 mg GAEs/100 g for fruits and leaves, respectively) and the polyphenol content in leaves was significantly higher than that in fruits for all samples (Figure 2). Conversely, mortiño fruits from Chimborazo showed significantly higher antioxidant capacity than those from Pichincha (12.84 ± 0.52 and 9.16 ± 0.41 mmol TE/100 g, respectively), but no significant differences were observed when comparing mortiño leaves obtained from both provinces. Additionally, no significant differences were observed when comparing the antioxidant capacity of fruit and leaf samples (Figure 3).

**Figure 4:**
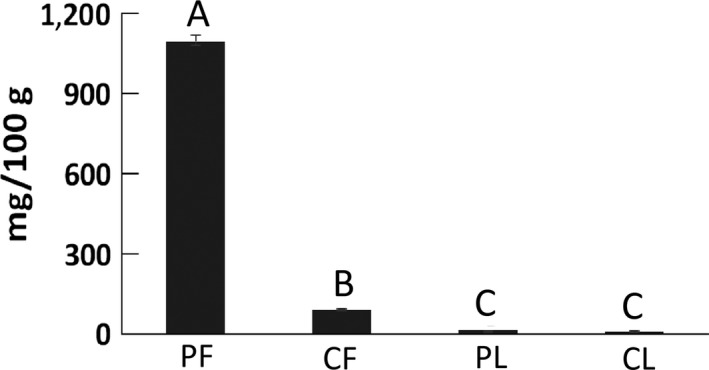
Concentration of anthocyanins in mortiño leaves and fruits from Pichincha (PF and PL) and Chimborazo (CF, and CL). Different letters (A, B, and C) above each column represent significant differences (*p* < .05) in the average anthocyanins concentration of each sample group

Fruit samples from Pichincha contained significantly higher average anthocyanins levels (1,095.39 ± 19.18 mg/100 g) than the fruit samples from Chimborazo (89.88 ± 0.65 mg/100 g), and anthocyanins levels in fruit were significantly higher than those in leaves for all samples (Figure 4).

### 3.3 Antibacterial properties

**Figure 5:**
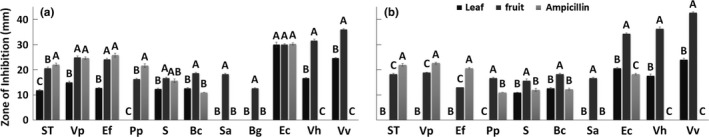
Zone of inhibition of mortiño leaf and fruit extracts as well as ampicillin on LB (a) or TSA (b) against *S*. Typhimurium (ST); *Vibrio parahaemolyticus* (VP); *Enterococcus faecalis* (EF); *Probionibacterium propionicum* (PP); *S. aureus* (S); *Burkholderia cepacia* (BC); *Pseudomona aeruginosa* (SA); *Burkholderia gladioli* (BG); *Escherichia coli* (EC); *Vibrio harveyi* (VH); *Vibrio vulnificus* (VV). Different letters (A, B, and C) for treatments in the same pathogen represent significant differences (*p* < .05)

All fruit extracts showed inhibition halos against the pathogens tested, and no growth inhibition was observed when ethanol alone was used. The highest ZOI observed was 40 mm and resulted from testing mortiño fruit extracts against *Vibrio vulnificus* in TSA, followed by 32 and 32 mm ZOIs of fruit extracts against *Vibrio harveyi* and *Escherichia coli* in TSA, respectively (Figure 5b). The highest average inhibition observed in LB was when using fruit extracts against *E. coli* and *V. vulnificus* with ZOIs of 26 and 18 mm, respectively (Figure 5a). Smaller average ZOIs were obtained with fruit extracts against *E. faecalis*,* P. propionicum*, and *S. aureus* in TSA with values of 10, 13, and 12 mm, respectively.

The culture media significantly affected the size of the ZOIs. The use of TSA yielded significantly higher inhibition of fruit extracts against *V. vulníficus*,* V. harveyi*, and *E. coli* with ZOIs of 40, 32, and 32 mm, respectively, when compared to the 32, 28, and 26 mm ZOIs obtained when using LB. Conversely, the use of LB yielded significantly higher inhibition of fruit extracts against *S*. Typhimurium, *Vibrio parahaemolyticus*, and *E. faecalis* with ZOIs of 16, 22, and 20 mm, respectively, when compared to the 14, 16, and 10 mm ZOIs observed when using TSA. Additionally, *P. aeruginosa*, considered a multiresistant bacteria (WHO, 2014), was only inhibited by mortiño fruit extracts with ZOIs of 12 mm in TSA and 14 mm in LB with no inhibition observed when using the antibiotic ampicillin (Figure 5a,b). No significant culture media effect was observed in the ZOIs of *P. propionicum*,* S. aureus*,* B. cepacia*, and *P. aeruginosa*. Similarly, mortiño leaf extracts caused a significantly greater inhibition of *S. aureus*,* V. parahaemolyticus*,* E. faecalis*, and *E. coli* on LB when compared to TSA. Leaf extracts also inhibited *S. aureus*,* B. cepacia*,* V. harveyi*, and *V. vulnificus* with no significant differences observed when comparing LB and TSA.

**Figure 6:**
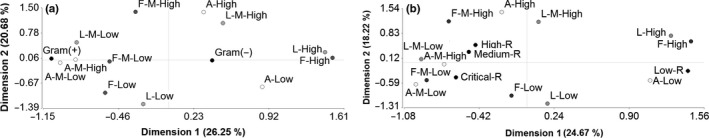
Correspondence analysis between Gram staining (a) or resistance class (b) vs inhibition halo size. Gram staining (●) was positive or negative, and resistance level (●) was low (Low‐R), medium (Medium‐R), high (High‐R), and critical (Critical‐R); leaf (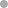), fruit (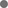), and ampicillin (○) halo sizes were low, medium–low (M‐Low), medium–high (M‐high), or high as indicated in Table 2

To perform the correspondence analysis, ZOIs were divided into low, medium–low, medium–high, and high halo sizes as determined by the 1st, 2nd, 3rd, and 4th quartile ranges, respectively. Table 2 shows the ranges obtained for leaf, fruit, and ampicillin inhibition. Correspondence analysis showed that the low and high ZOIs grouped with Gram‐positive and Gram‐negative bacteria, respectively (Figure 6). Similarly, low and medium–low ZOIs grouped with the medium, high, and critical potential of antibiotic resistance, whereas high ZOIs grouped with the low potential of antibiotic resistance (Figure 6b).

**Table 2:** Range categories of zone of inhibition diameters (ZOIs) obtained by in vitro tests against all the pathogens described in Figure 5

Range category^a^	Leaf extract (mm)	Fruit extract (mm)	Ampicillin (mm)
Low	0.0	Below 14.0	0.0
Medium–low	0.2–10.0	14.2–17.0	0.2–11.0
Medium–high	10.2–13.8	17.2–25.0	11.2–21.0
High	14.0–30.0	25.2–34.0	21.2–28.0

a Obtained by calculating the 1st, 2nd, 3rd, and 4th quartiles.

## 4 DISCUSSION

Sequences of the matK DNA region of mortiño plants were similar among all samples, except for the samples from the Cotopaxi province that showed one polymorphic nucleotide and grouped separately in the phylogenetic tree. Mortiño plants growing in Cotopaxi were mostly located near the Quilotoa volcano while plants from Pichincha and Carchi were from populated rural areas. Results are in agreement with previous studies in which genetic variation of *Vaccinium* spp. correlated with differences in climate and geography (Borsch & Quandt, 2010). Similarly, highly heterogeneous climatic conditions have been responsible for variations in populations of *Vaccinium meridionale* in Colombia (Ligarreto, Patiño, & Magnitskiy, 2011), and a decrease in *Vaccinium* spp. leaf size proportional to the increase in geographical altitude has been observed (Cecco et al., 2016).

Sequences of the rcbL region were homogeneous across all sampled provinces, and no polymorphism was observed. The rbcl DNA region has shown lower variability than the matK region in various plant species (Funke, 2016). In this study, polymorphism was only found in matK sequences probably due to the potential of this region to evolve more quickly than other DNA regions (Wicke & Quandt, 2009). Prior to this study, no rcbL sequences of mortiño could be found in available databases.

Various methods are available to determine the antioxidant capacity of plant products including ABTS, 2,2‐diphenyl‐1‐picrylhydrazyl, oxygen radical absorption capacity, ferric reducing antioxidant power, among others. Applications in *Vaccinium* spp. (Connor, Luby, & Tong, 2002; Oszmiański, Kolniak‐Ostek, Lachowicz, Gorzelany, & Matłok, 2017) and other fruits such as guava (Thaipong et al., 2006) have yielded comparable results across the different antioxidant capacity methods. Slightly higher antioxidant capacity values were observed in *Vaccinium* spp. when using ABTS in a recent study (Rodrigues et al., 2011). In this study, ABTS was selected as the method for assessing the antioxidant capacity in mortiño.

The total polyphenol content of mortiño leaves was similar to the 255 mg GAEs/100 g reported for blueberry leaves in previous studies (Venskutonis et al., 2016), but the total polyphenol content and antioxidant capacity of mortiño fruits were significantly lower than the values reported for the fruits of other berries. Fruits of *V. corymbosum* showed polyphenol levels of up to 528.15 mg GAEs/g and antioxidant capacity of up to 90.07 mmol/L Fe^2+^/100 g (Dragović‐Uzelac et al., 2010). However, the antioxidant levels of mortiño fruits observed in this study can be considered as high in comparison with other antioxidant‐rich foods such as nuts, chocolate, and vegetables (Carlsen et al., 2010).

Mortiño fruits showed significantly higher anthocyanin levels than the 376.2 mg/100 g observed in *V. floribundum* fruits analyzed in previous studies (Prencipe et al., 2014). Additionally, the levels of anthocyanins in fruits were significantly greater than those in leaves, but the levels of polyphenols were significantly higher in leaves when compared to fruits. Results are in agreement with previous reports where anthocyanins were observed in fruits of *Vaccinium* spp. and not in leaves (Feng et al., 2017), but phenolic acids and flavonols were significantly higher in leaves when compared to fruits (Teleszko & Wojdyło, 2015).

Significant differences were observed in the polyphenols, antioxidant, and anthocyanin contents of fruits from Pichincha and Chimborazo, suggesting an environmental effect in the formation of these compounds. The formation of polyphenols has been strongly associated with environmental conditions, probably due to specialization reactions occurring under specific conditions of radiation, altitude, and temperature (Goyali, Igamberdiev, & Debnath, 2013) as well as soil composition (Dragović‐Uzelac et al., 2010).

Fruits from Pichincha showed significantly higher levels of polyphenols and anthocyanins but significantly lower antioxidant capacity than fruits from Chimborazo. A positive correlation was observed between polyphenols and anthocyanins, whereas a negative correlation between the antioxidant capacity and polyphenol levels was detected. Positive correlations between anthocyanin and polyphenol contents have previously been observed (Prencipe et al., 2014), but negative correlations between polyphenols and antioxidant capacity are not in agreement with recent findings that suggest a strong positive correlation between both compound classes in fruits and leaves of other species of *Vaccinium* (Meriño‐Gergichevich et al., 2015). However, negative correlations between polyphenols and antioxidant capacity have been observed in other fruits like guava (Vasco, Ruales, & Kamal‐Eldin, 2008). Further research is needed to identify the compounds contributing to the antioxidant capacity mortiño fruits and leaves.

Significant inhibitory effects of mortiño leaf and fruit extracts were observed against all pathogens tested. In particular, the 16, 18, 32, and 20 mm ZOIs observed in mortiño fruit extracts against *S. aureus*,* P. aeruginosa*,* E. coli*, and *S. *Typhimurium (Figure 5) where higher than those reported for other superfruits. Extracts of the aril of pomegranate showed average ZOIs of 23.7 and 9.71 mm against *E. coli* and *S. *Typhimurium, respectively (Nuamsetti, Dechayuenyong, & Tantipaibulvut, 2012) while extracts of the pomegranate fruit inhibited the growth of *S. aureus*,* E. coli*, and *P. aeruginosa* with ZOIs of 2.3, 0, and 6.0 mm, respectively (Opara, Al‐Ani, & Al‐Shuaibi, 2009). Similarly, extracts of quince pulp showed ZOIs of 16.2 and 8.2 mm against *P. aeruginosa* and *E. coli*, respectively (Sami Fattouch et al., 2007).

The ZOI values obtained by mortiño leaf extracts against *S. aureus* (12 mm) are similar to those observed in other plant extracts, but inhibition of *E. coli* and *V. parahaemolyticus* (29 and 15 mm ZOI, respectively) by mortiño extracts (Figure 5) was higher than those reported in the literature. However, mortiño leaves did not inhibit *P. aeruginosa*. Leaf extracts of *Merremia emarginata* (12.0, 12.0, and 9.0 mm ZOIs) and *Cassia fistula* (16.0, 17.0, and 16.0 mm ZOIs) inhibited *S. aureus*,* E. coli*, and *P. aeruginosa*, respectively (Bhalodia & Shukla, 2011; Elumalai, Ramachandran, Thirumalai, & Vinothkumar, 2011). Similarly, the extract of guava leaves did not inhibit *E. coli* but showed a 11 mm ZOI against *S. aureus* (Biswas, Rogers, McLaughlin, Daniels, & Yadav, 2013). Additionally, neem leaf extracts inhibited the growth of *S. aureus* and *V. parahaemolyticus* with ZOIs of 10 mm for both bacteria (Farjana, Zerin, & Kabir, 2014).

The observed antimicrobial activity is probably due to the high‐polyphenol levels in the extracts. Phenolic compounds containing hydroxyl groups have been reported to inhibit the enzymatic activity of microorganisms (Samad, Debnath, Ye, Hasnat, & Lim, 2014). Additionally, phenolic acids such as gallic and caffeic acids are potential inhibitors of proline metabolism in pathogens such as *L. monocytogenes* (Apostolidis, Kwon, & Shetty, 2008), and phenolic compounds can be complexed with proteins in the outer membrane of microorganisms (Yow, Tang, Chu, & Huang, 2012), potentially resulting in the pathogen's inhibition or death.

Mortiño leaf extracts did not inhibit the growth of *P. propionicum* and *S. aeruginosa* on any culture medium tested, and inhibition of *S*. Typhimurium, *V. parahaemolyticus*, and *E. faecalis* was observed on LB agar only. Results are in agreement with previous reports suggesting poor inhibition effects of plant extracts when tested on TSA (Shen et al., 2014).

Various culture media including TSA, Nutrient Agar (Pervin et al., 2013), Tryptic Soy Broth (Shen et al., 2014), Potato Dextrose Agar (Viskelis et al., 2009), among others have been used to test the inhibitory effect of *Vaccinium* spp. extracts. However, the effect of the culture media on the inhibition halos caused by *Vaccinium* spp. extracts has not been evaluated. In this study, the culture media significantly affected the size of mortiño's ZOIs produced against *V. vulnificus*,* V. harveyi*,* E. coli*,* S*. Typhimurium, *V. parahaemolyticus*,* E. faecalis*, and *P. aeruginosa*. Results are in agreement with previous reports showing a significant influence of the culture media on the size of ZOIs caused by plant extracts against bacterial pathogens (Huys, D'Haene, & Swings, 2002).

Results from the correspondence analysis suggest that Gram‐negative pathogens are more susceptible to mortiño leaf and fruit extracts than the Gram‐positive ones. The finding of effective antimicrobials against Gram‐negative bacteria has been challenging due to the low permeability of the outer membrane in this type of microorganisms (Delcour, 2009). Mortiño leaves and fruits have the potential to become the source of new and effective antimicrobials that can be used in the food and pharmaceutical industries.

Correspondence analysis grouped the ZOI sizes with the expected antibiotic resistance as reported by the WHO (WHO, 2014). Low ZOI sizes grouped with highly resistant strains and vice versa. Results support WHO report that suggests that the pathogens tested possess innate mechanisms of resistance to broad‐spectrum antibiotics, including ampicillin and other natural antimicrobials (WHO, 2014).

## 5 CONCLUSIONS

Mortiño leaves and fruits contain high levels of antioxidants, polyphenols, anthocyanins, and antimicrobial activity. Mortiño has the potential to become the source of effective bioproducts that can be used in the food and pharmaceutical industries to develop novel nutraceuticals and antimicrobials.

## CONFLICT OF INTEREST

The authors have no conflict of interest to report.

## ETHICAL REVIEW

This article does not contain any studies with human or animal subjects.
